# Primary Stabbing Headache in Children and Adolescents

**DOI:** 10.3390/life14020216

**Published:** 2024-02-02

**Authors:** Maria Reimers, Ilaria Bonemazzi, Francesco Brunello, Elena Cavaliere, Stefano Sartori, Irene Toldo

**Affiliations:** Juvenile Headache Center, Department of Woman’s and Child’s Health, University of Padua, 35128 Padua, Italy

**Keywords:** primary stabbing headache, other primary headaches, children, adolescents

## Abstract

Background: Primary Stabbing Headache (PSH) is characterized by brief, focal, and paroxysmal pain (“stab”), occurring sporadically or in clusters. Data on pediatric cases are poor. Methods: We performed a comprehensive literature review by searching PubMed, Cochrane, and Embase in order to collect pediatric case reports and case series of PSH. Results: A total of 12 out of 162 articles assessed for eligibility were finally included. The prevalence of PSH and probable PSH varies from 2.5 to 10% among children with primary headaches and it is higher among children aged less than 6 years old. The mean age of onset is between 7 and 11 years of age. Attack duration greatly varies, ranging from a few seconds to several minutes. The intensity of pain is usually from moderate to severe. Associated symptoms are infrequent but may be observed (mainly photophobia, vertigo, nausea, and vomiting). Neuroradiological findings are usually unremarkable; EEG may show sporadic epileptiform abnormalities (up to 30% of cases). Preventive therapy is anecdotal, including treatment with indomethacin, trazodone, valproate, and amitriptyline. Conclusion: PSH is a common but still underdiagnosed entity among children with primary headaches; further and larger cohort studies are needed to better assess, in particular, prognosis and response to therapy.

## 1. Introduction

Primary Stabbing Headache (PSH) is characterized by brief, focal, and paroxysmal pain (“stab”), which can manifest with extreme variability in frequency over time, sporadically, or in clusters [1]. Reports of a strong family history of PSH are lacking, contrary to other primary headache disorders (such as migraine and tension-type headache). The genetic background of PSH is still unknown. 

Pathogenesis of PSH has been postulated, given the peculiar characteristics of pain: hyperexcitability and spontaneous firing of cervical and trigeminal nerves (which provide sensitive innervation of the scalp), due to both central and peripheral sensitization mechanisms, could play a major role [2]. However, this hypothesis lacks the support of experimental studies. Coexisting autoimmune pathologies that could determine focal demyelination of sensory nuclei in the brainstem (multiple sclerosis, autoimmune vasculitis, antiphospholipid syndrome) have been inconsistently described among adult patients with PSH, while this association has not been noted among pediatric cases [3]. 

Differential diagnosis requires promptly excluding secondary causes of headache (neoplastic and vascular etiologies), especially if the localization of pain is fixed or unusual. Other types of primary headache should be ruled out considering pain duration (paroxysmal hemicrania), pain localization (trigeminal neuralgias), presence/absence of accompanying cranial autonomic symptoms (trigeminal autonomic cephalalgias), presence/absence of allodynia/dysesthesia at the level of the scalp (occipital neuralgia), and presence/absence of specific trigger factors (exertional and cold stimulus headaches).

Despite its relatively high prevalence in the pediatric population, especially among young children, data about pediatric PSH are few and poorly standardized. This could be related to the strict diagnostic criteria and the frequent coexistence of other primary headaches, which could make the diagnosis and the recruitment of patients in large cohort studies more difficult.

In this study, we performed a narrative literature review searching for case reports and case series in order to better define the prevalence, clinical characteristics, treatment, and prognosis of pediatric patients with PSH.

## 2. Classification and Diagnostic Criteria

Stabbing-type headache was first mentioned in the 1988 International Classification of Headache Disorders (ICHD-1) [4]. The category was named “Idiopathic Stabbing Headache” and it was included in the miscellaneous headaches without structural lesions. Idiopathic Stabbing Headache was described as a transient stabbing pain lasting from fractions of a second to several seconds, occurring in the absence of organic disease of the underlying structures or cranial nerves. 

In 2004, a revised version of the ICHD (ICHD-2) was published in which the category “Idiopathic Stabbing Headache” was replaced by “Primary Stabbing Headache”, making its first appearance in the International Classification [5]. The absence of accompanying symptoms was required to make a diagnosis.

Interestingly, in the first two versions of ICHD, one of the main criteria for the diagnosis of stabbing-type headache was the localization of the pain to the distribution area of the first division of the trigeminal nerve (orbit, temple, and parietal area). The association with migraine and a positive response to indomethacin were included in the comment section. 

In the latest version of the ICHD published in 2018 (ICHD-3), the diagnosis of PSH requires head pain occurring as a single stab or series of stabs lasting up to a few seconds, occurring with irregular frequency and without cranial autonomic symptoms [1]. Of note, this revised version does not include pain localization as the main criterion, specifying in the comment section that up to 70% of cases involve extra-trigeminal regions. Furthermore, differently from the previous version, in ICHD-3 not all accompanying symptoms (such as nausea, vomiting, photophobia, or phonophobia) must be excluded, but only cranial autonomic symptoms [6]. Table 1 outlines the current diagnostic criteria. 

Moreover, in the ICHD-3 a new diagnostic entity was described: “probable PSH”. Diagnostic criteria are outlined in Table 2.

## 3. Materials and Methods

We searched PubMed, Cochrane, and Embase with the following queries: “Primary Stabbing Headache”, “Idiopathic Stabbing Headache”, “Stabbing Headache”, “Ice-Pick Headache” and “PSH”. We included all full-text articles, published from 1991 to October 2023. A total of 162 full-text articles were assessed for eligibility. 

We manually screened all articles, searching for case reports and case series of patients with Primary Stabbing Headache (PSH) and probable PSH, according to ICHD-3 criteria. Seventy-eight articles were excluded because they reported data not significant to our purpose. We restricted our search to articles including pediatric patients (age range 0–18 years) who respected diagnostic criteria for PSH, therefore excluding 63 studies that reported data on the adult population. Two articles were found twice when searching in different engines. We then analyzed 20 studies focusing on PSH in the pediatric population, with the goal of describing the following features: epidemiology, clinical manifestations, data from investigations, and treatment. Out of these, 7 studies were excluded because they contained mixed adult and pediatric cohorts without stratified data on the pediatric population. We finally included 12 studies on which our comprehensive literature review was conducted [7,8,9,10,11,12,13,14,15,16,17,18]. 

Based on the different methodologies employed by the considered studies, it was not possible to conduct a systematic review or meta-analysis with the available data.

## 4. Prevalence

Previous studies show that the prevalence of PSH and probable PSH, among pediatric patients with primary headache, varies from 2.5 to 9.97% [16,18], confirming that PSH is a common entity among children and adolescents. Data available in the literature are insufficient to estimate an overall prevalence among the pediatric population.

## 5. Mean Age of Onset

The mean age of onset is between 7 and 11 years of age [7,18]. A recent study by Saygi found that 16.9% of patients with PSH had clinical onset before 6 years of age [18]. Some cases in which the onset occurred as early as 2 years of age have also been described [4,9]. In a study cohort conducted by Raieli et al. in 2002, the mean age of onset among girls was 10.8 years (range, 2.6–17.8), while it was 8.8 years (range, 5.0–13.0) among boys [8]. Differently, Saygi found that the mean age of onset among girls was 10.1 years (SD ± 3.7), while it was 11.4 years (SD ± 3.4) among boys, not showing a statistically significant difference between genders [18].

A retrospective study by Raieli et al. analyzed the frequency of primary headache subtypes in a population of 105 cephalalgic children below 6 years of age; interestingly, the prevalence of PSH (12.4%) was significantly higher in the study cohort compared with a population of 100 cephalalgic children older than 6 years of age (3%) [11]. These data suggest that PSH prevalence could be higher among young children.

## 6. Gender Distribution

The gender distribution among children with PSH greatly varies. In most studies, males and females were roughly equally represented [7,10,12,13,16]. In the studies by Raieli et al. and Saygi, a higher prevalence among females was reported with an F:M ratio of, respectively, 2:1 and 1.5:1, which is in line with data from studies conducted on the adult population [8,18].

## 7. Presence of Other Headache Disorders

Moreover, the overlapping presence of other headache disorders was also taken into consideration by some authors. Ahmed et al. found that 12 patients out of 42 (29%) presented other headache disorders, mostly migraine, which is in line with the ICHD-3 comments on PSH [16]. Contrarily, in the study by Fusco et al., no previous history of other types of headache was reported among patients [10]. 

In the case study presented by Hofstadter-Duke et al., the patient was diagnosed with two additional types of primary headaches, other than PSH, including tension-type headache, occurring daily, and migraine, occurring with a weekly frequency [14]. The patient identified Stabbing Headache, which occurred daily, as the most debilitating type of headache pain.

## 8. Family History of Headache Disorders

Most studies also analyzed the prevalence of a family history of headache disorder, finding it positive in 31–58% of patients, mainly represented by a positive history of migraine: in the case study by Takeshita et al., three out of five children (60%) also manifested migraine and had a relative who suffered from migraine [17]. The study by Ahmed et al. found that only 2 patients out of 42 (5%) had a relative who suffered from PSH [16]. 

## 9. Clinical Features

Clinical features that emerged from the studies we analyzed are summarized in Table 3.

### 9.1. Type of Pain, Intensity, and Localization

According to the ICHD-3 definition of PSH, pain is characterized by a single stab or series of stabs. Therefore, the pain is most often described by patients as having a stabbing quality. However, in one study by Saygi up to 34% of patients reported a “throbbing” type of pain [18]; this may be due to the fact that in the pediatric population, it is more difficult to precisely describe the pain, but still the duration pattern and the associated symptoms were highly suggestive of PSH. 

The intensity of pain ranges usually from moderate to severe, even if some patients with PSH in the studies by Soriani et al. and Saygi described mild to moderate pain, overall not affecting daily activities [7,18]. 

Pain is often described as unilateral [7,9,11,12,16] and it is most often localized in the frontal region [7,8,16,18]. Occipital pain is also described among pediatric patients with PSH [13].

### 9.2. Duration and Frequency

The attack duration is undoubtedly the most difficult criterion to meet to make an ICHD-3 diagnosis of PSH because of its strict temporal range. In our experience, it is particularly challenging when interviewing pediatric patients because young children have difficulties distinguishing a single stab from a series of stabs. According to the ICHD-3 definition, each stab should last for up to a few seconds, specifying that most studies (on the adult population) report stabs that last 3 s or less and rarely for 10–120 s [1]. This limitation highlights the importance of “possible PSH”, a diagnostic category introduced in the ICHD-3; “possible PSH” can be diagnosed even without meeting the duration criteria.

Considering the pediatric population, a remarkable variability in attack duration emerged when comparing different studies from the literature, ranging from a fraction of a second to several minutes. Raieli et al. reported that in most PSH attacks, pain lasts a few seconds, but 20% of patients reported some attacks lasting several minutes [8]. Fusco et al. reported a mean duration of 5 min [10]. Moreover, Ahmed et al. reported that some patients (23%) had symptoms lasting up to 15 min [16]. These patients, according to the definition of ICHD-3, would not fall under the diagnosis of PSH; however, as they do not fall under any other type of headache and meet the other criteria of PSH, they can be considered as “probable PSH” [1].

Concerning the duration of attacks, Fusco et al. reported a mean duration of 5 min, which reached 15 min in the Saygi et al. study [10,18].

All studies investigated the frequency of attacks, which is more than once per week in most cases [7,8,10,11,16,18]. A monthly frequency was reported in 15 to 37% of patients [8,16]. 

### 9.3. Associated Symptoms 

Overall, in all studies, most patients did not report any accompanying symptoms. When complained, the most common symptoms were: nausea (7–14.3%), photophobia and/or phonophobia (2.6–19%), vertigo (1.3–8%), and vomiting (1.3–5%) [7,16,18]. Soriani et al. reported a high frequency (in 47% of patients) of periodic syndromes: mainly cyclic vomiting and recurrent abdominal pain, preceding the PSH onset [7]. Fusco et al. noticed that motion sickness was present in 8/23 subjects and vertigo in only one patient independently of the headache [10]. In the study by Vieira et al., patients had no associated signs or symptoms apart from slight pallor probably due to a vagal reaction (two out of 17 cases) [11]. Furthermore, a retrospective clinical study by Raieli et al. reported that headaches are associated with sudden painful grimaces (numbers not specified) [11].

### 9.4. Children vs. Adults

To better characterize PSH among children and adolescents, we compared the pediatric studies included in our review with data from recent studies on the adult population, selecting some of the most recent reviews, aiming to find potential differences in clinical presentation between pediatric and adult patients. Stabbing quality of pain with variable intensity (mostly from moderate to severe) is described among adult PSH patients [19,20], which is in line with what we found in the pediatric population. As reported for children, adults also present unilateral pain in most cases [19,20,21,22,23]. Differently from our data, localization is often described in the occipital region among adult patients [19,21,22]. Duration of pain is usually less than or equal to three seconds [19,21], and only in a minority of adult cases up to 60 s [22]. These findings differ from what is reported in pediatric cases since long-lasting PSH attacks, sometimes up to 15 min, are described. Regarding associated symptoms, studies on adults report the presence of jolts (which are sudden movements such as grimacing or shrugging) following the jabs, body jabs, and allodynia as relatively common clinical findings [19,21,22]. A clinic-based study by Fuh et al. also reported the presence of vocalization in 18% of patients [22]. Interestingly, these symptoms are rarely reported in children: only Raieli et al. described a painful grimace occurring with a headache [8]. Accompanying symptoms such as nausea or vomiting, dizziness, and photophobia/phonophobia during attacks have similar frequency among children and adults [22,23,24].

## 10. Triggering Factors

Six out of the twelve selected papers deal with triggering factors in pediatric PSH.

In most studies, no precipitating or triggering factors were clearly identified [11,13,15,18]. Soriani et al. found a psychogenic triggering event in 18 of 83 patients (22%) [7]. In the population studied by Ahmed et al., 7.1% of patients (*n* = 3) reported attacks triggered by exercise (*n* = 1) or stress (*n* = 2). The authors considered alternative diagnoses for these patients, such as primary exercise headache, but they did not match the required diagnostic ICHD-3 criteria regarding the frequency and features of headache attacks [16].

Studies in the adult population have found a precipitating factor in up to 50% of patients, particularly stress, recent illness, extreme weather conditions, or sleep disturbance/fatigue [2]. Interestingly, in the adult population, possible infectious triggers have been identified; for example, a case report described an adult patient who developed several attacks of PSH after COVID-19 infection [25]. No such finding was reported in the latest studies we analyzed.

## 11. Neuroradiological Findings

Seven out of twelve analyzed articles provide neuroradiological data about pediatric PSH.

Brain imaging, mainly performed as magnetic resonance imaging (MRI), was normal in almost all patients [7,11,13,15,16,18]. Fusco et al. reported one patient with cerebellar vermis hypoplasia with mild ventricular dilation [10]. In another study, radiological evidence of sphenoidal or ethmoidal sinusitis was detected (in 3/17 patients) in the absence of symptoms or signs of acute respiratory infection during periods of headache [11]. In the Mukharesh et al. study, a detailed vasculitis workup, MRI, and magnetic resonance venography were performed in one patient, and all results were within normal limits [13].

Considering studies on the adult population, similar data were found regarding the negativity of radiological examinations: many authors agree that, as in other primary headaches, radiological investigations should be performed to consider and rule out possible secondary causes of secondary aetiologies [26,27,28,29]. If PSH attacks have atypical features (such as longer duration, substantial background pain, or other red flags), computed tomography, or MRI of the brain or cervical spine is mandatory, especially in children under 6 years of age [2,6,26].

## 12. Electroencephalographic (EEG) Findings

The diagnostic value of EEG is unclear and its use for the diagnosis or differential diagnosis of PSH is generally believed to be unnecessary [26].

Seven out of twelve selected articles provide EEG data about pediatric PSH.

EEG recordings were typically normal [7,13]. In some studies, interictal epileptiform abnormalities were reported (4–40% of cases) [8,10,11,18]. In particular, in the cohort studied by Vieira et al., EEG was performed in 5/17 patients: three were normal, one had epileptiform abnormalities in the occipital region, and one in the frontal region [9]. In one of the studies, occasional posterior slow waves of high amplitude were observed in 24% of the cases, with marked slowing during hyperventilation [10]. A similar result was found by Raieli et al. (2002): epileptiform abnormalities (focal sharp waves, spikes, slow waves, and rolandic spikes) were observed in 20% of the performed EEGs (*n* = 6 patients). Of these patients, one reported focal epilepsy and intellectual disability, another had suffered a mild head injury about thirty days earlier, another patient had mild psychomotor retardation, and another one had a family history of epilepsy [8]. In another study, EEG abnormalities were found in 30.7% of PSH patients, but the difference with other primary headaches was not statistically significant [11].

It is difficult to interpret EEG data in PSH; in our opinion, it is not possible to state if these could be relevant for the diagnosis since not all patients systematically underwent electroencephalographic investigation.

## 13. Treatment

Five out of twelve analyzed studies provide findings about prophylaxis therapies in pediatric PSH and none provide findings about on-demand therapies. Concerning prophylaxis therapy, Mukharesh et al. 2011 found that most of the patients did not receive any treatment (3/5), and two patients were treated with amitriptyline (a single dose of 10–25 mg at bedtime) because of their frequency of stabbing pain headaches and associated migraine headaches, with significant improvement within the first months of treatment [13]. In the Raieli et al. study, seven female patients (23%) were selected for prophylactic therapies because of frequency (at least weekly) and severity of PSH attacks or because of other coexisting primary headaches requiring preventive treatment. Indomethacin (75 mg/day, in two patients), trazodone (0.5 mg/kg, in one patient), and L-5 hydroxytryptophan + riboflavin (100 mg + 100 mg, in two patients) were used with a reduction of more than 50% in headache attacks. One patient, suffering from 40–50 attacks per day, treated with carbamazepine (10 mg/kg/day) for two months achieved a total resolution of PSH attacks. In one patient treated with flunarizine (5 mg/day), no changes in the frequency or intensity of PSH attacks were observed [8]. Another study considered the possibility of prophylaxis through melatonin. Half a tablet of 1.5 mg of melatonin was administered daily (0.07 mg/kg) at night; this therapy showed a significant reduction in headache frequency within the first two weeks of treatment. In addition, during a follow-up of six months, no new episodes were documented [15]. A single controlled case study suggests that external hand warming could reduce the intensity and frequency of PSH attacks with a significant increase in pain-free days; the patient’s caregiver also reported overall better functioning in daily life. External warming consists of applying warm mittens for 5 min two times a day for one month, which turned out to be a noninvasive procedure with no side effects [14]. No specific acute therapy has been described in the selected articles focusing on children and adolescents with PSH; this gap in the literature could be due to the overall brief duration of each single PSH attack [17,26]. 

Data available emerge from studies on the adult population. The most effective abortive treatment is indomethacin, with a good response in up to 60% of patients [6]. Indomethacin is not FDA-approved for children younger than 15 years; however, some studies have been published recommending a trial of indomethacin in pre-adolescents with PSH, in which secondary headaches have been carefully ruled out [30]. In case of inefficacy, poor tolerability, or contraindications to indomethacin, other treatment options have been tested but their efficacy is difficult to assess, due to the lack of a large cohort of patients and controlled studies. These treatment options include topiramate, acetazolamide, melatonin, and other NSAIDs, such as etoricoxib and celecoxib, onabotulinumtoxin A, gabapentin, and nifedipine [6,26,31].

## 14. Prognosis

Regarding the prognosis of pediatric PSH, few and incomplete data were found only in four out of twelve analyzed studies. 

A recent review on PSH in the adult population distinguishes the clinical course of PSH into monophasic, intermittent, or chronic daily, finding the intermittent course, characterized by infrequent episodes of sporadic or clustered stabbing pain, as the predominant one [26].

Unfortunately, data on the pediatric population are not sufficient to make a clear distinction between different clinical courses like in the adult population.

The data we found concerning children were mostly favorable, indicating that at follow-up most patients show a general clinical improvement [7,11,13]. The prospective study by Ahmed et al. found the chronic–intermittent course to be the dominant one (38 out of 42 patients) [16].

## 15. Conclusions

PSH is a relatively common but still underdiagnosed entity among children and adolescents. Clinical features of PSH attacks are similar both in children and adults, with the main exception being the duration of stabs, which tends to be more long-lasting in pediatric cases. Data regarding the natural history and evolution over time of pediatric-onset PSH are still lacking. Further cohort studies are needed in the pediatric population to better assess, in particular, prognosis and response to therapy.

## Figures and Tables

**Table 1:** ICHD-3 diagnostic criteria of PSH [1].

Primary Stabbing Headache
A. Head pain occurs spontaneously as a single stab or series of stabs and fulfilling criteria B and C. B. Each stab lasts for up to a few seconds ^1^. C. Stabs recur with irregular frequency, from one to many per day ^2^. D. No cranial autonomic symptoms. E. Not better accounted for by another ICHD-3 diagnosis. Notes: ^1.^ Studies show that 80% of stabs last 3 s or less; rarely, stabs last for 10–120 s. ^2.^ Attack frequency is generally low, with one or a few per day. In rare cases, stabs occur repetitively over days, and there has been one description of status lasting one week.

**Table 2:** ICHD-3 diagnostic criteria of probable PSH [1].

Probable Primary Stabbing Headache
A. Head pain occurring spontaneously as a single stab or series of stabs B. Two only of the following: 1. each stab lasts for up to a few seconds 2. stabs recur with irregular frequency, from one to many per day 3. no cranial autonomic symptoms C. Not fulfilling ICHD-3 criteria for any other headache disorder D. Not better accounted for by another ICHD-3 diagnosis

**Table 3:** Clinical features of PSH according to the studies we found.

Study (*n* of Patients)	Quality of Pain	Side	Localization	Duration	Intensity	Frequency
Soriani et al., 1996 [7] (*n* = 83)	Stabbing 100%	Bilateral 48% Unilateral 30% Alternating 22%	Periorbital/frontal 49% Parieto-occipital 23% Temporal 20% Vertex 8%	Sec to several min 100%	Severe 30% Moderate 30% Mild 40%	Once/week 21% >Once/week 52% Monthly 27%
Raieli et al., 2002 [8] (*n* = 30)	Stabbing 100%	Bilateral 30% Unilateral 63.3% Central 6.6%	Temporal 36.7% Frontal 26.7% Vertex 13.3% Parietal 10% Occipital 3.3% Nasal 3.3% Variable/multiple 23.3%	<2 s 53.3% >2 s to several min 46.6%	Intolerable 20% Moderate-severe 63.3% Mild 16.6%	Daily 46.7% Weekly 16.7% Monthly 36.7%
Evans et al., 2002 [9] (*n* = 1)	Stabbing	Unilateral alternating	Temple	5 s–2 min	ns	1–4 times/day
Fusco et al., 2003 [10] (*n* = 23)	Stabbing 100%	Bilateral 60% Unilateral 40%	ns	<3 min 47% >3 min 53%	Severe 57% Moderate 30% Mild 10%	Once/week 35% >Once/week 65%
Raieli et al., 2005 [11] (*n* = 17)	Stabbing 100%	Bilateral 23.5% Unilateral 76.5%	Frontotemporal 55.5% Vertex 17.6%	1–10 s 100%	Severe 35.3% Moderate 64.7%	>1/day 58.9% >1/week 41.1%
Vieira et al., 2006 [12] (*n* = 17)	ns	Bilateral 12% Unilateral 70% ns 18%	Occipital 53% Frontal 35% Temporal 6% Parietal 6%	<1 s 18% 1 to 5 s 71% Ns 11%	Severe 94% Moderate 6%	Daily 29% Weekly 47% Monthly 24%
Mukharesh et al., 2011 [13] (*n* = 5)	Sharp	ns	Temporal 60% Occipital 20% Orbital 20%	<3 s 100%	Severe	Daily 20% Weekly 20% Monthly 60%
Hofstadter-Duke et al., 2011 [14] (*n* = 1)	Stabbing	ns	ns	15–20 s	Severe	1–2 times/day
Salazar et al., 2018 [15] (*n* = 1)	Stabbing	No predominant laterality	Temporal	3–4 s	Mild to moderate	Weekly
Ahmed et al., 2020 [16] (*n* = 42)	Stabbing 100%	Bilateral 36% Unilateral 60% Unilateral and/or bilateral 4%	Temporal 14% Frontal 29% Vertex 2% Parietal 26% Occipital 17% Parieto-occipital 2% Retro-orbital 2% Variable 7%	≤3 s 17% ≤ 2 min 19% >2 min 64%	Severe 90% Moderate to severe 10%	Daily 48% Daily to weekly 17% Weekly 21% Monthly 14%
Takeshita et al., 2021 [17] (*n* = 5)	Stabbing 100%	ns	Temple 83% ns 17%	ns	Variable 100%	ns
Saygi, 2022 [18] (*n* = 77)	Stabbing 10.4% Sharp 6.5% Compressing 9.1% Throbbing 33.8% Indescribable 40.3%	Bilateral 68.8% Unilateral 19.5% Unilateral or bilateral 11.7%	Temporal 23.4% Frontal 54.5% Occipital 11.7% Multifocal 10.4%	Seconds 37.7% 1–5 min 19.5% 1–10 min 24.7% 1–15 min 18.2%	Mild to moderate 100%	Daily 46.8% Weekly 29.9% Monthly 23.4%

ns = not specified.

## Data Availability

The data of the current study (not included in this published article) are available from the corresponding author upon reasonable request.
